# The role of chronotype in the interaction between the alerting and the executive control networks

**DOI:** 10.1038/s41598-020-68755-z

**Published:** 2020-07-17

**Authors:** Víctor Martínez-Pérez, Lucía B. Palmero, Guillermo Campoy, Luis J. Fuentes

**Affiliations:** Facultad de Psicología, Universidad de Murcia, Campus de Espinardo, 30100 Murcia, Spain

**Keywords:** Psychology, Human behaviour

## Abstract

Chronotype refers to the time of day preferred by individuals to perform daily activities according to their circadian rhythm. We asked whether synchrony effects, that is, the difference in performance between the optimal and non-optimal time of day as a function of chronotype, are observed in two tasks that differently involve the endogenous component of the alerting network, the psychomotor visual task (PVT) and the flanker task. From an initial sample of 132 students that filled in the Morningness–Eveningness Questionnaire (MEQ), 18 were classified as Morning-types and 16 as Evening-types. Evening-types showed synchrony effects in both tasks, whereas Morning-types failed to show synchrony effects in the flanker task and when the PVT was first performed at the non-optimal time of day. Thus, Morning-types might have seen increased their vigilant attention at their non-optimal time of day due to the cognitive demands of the flanker task and to the novelty with the PVT. Phasic alerting generated by alerting tones increased conflict score in the flanker task, but time of day did not modulate the congruence effect. Chronotype determines vigilant attention more decisively in Evening-types than in Morning-types individuals. Also, exogenous but not endogenous alerting exerts a deleterious effect on conflict resolution.

## Introduction

In dealing with daily activities, organisms need to reach an appropriate level of arousal (activation) to perform efficiently, which varies according to the famous Yerkes–Dodson’s inverted-U shaped law^1^. According to this law, the optimal level of arousal very much depends on task difficulty, so that performance on rather easy tasks profits from high levels of arousal whereas low levels of arousal are beneficial for rather difficult tasks. In other words, depending on its difficulty, each task requires an optimal level of arousal, with important decrements in performance when such level deviates, below or beyond, from the optimal one.

In Posner and Petersen’s^2^ theory, such arousal state is linked to the alerting network of the attention system, which is involved both in achieving and maintaining an optimal level of activation, and in preparing the person to perceive and/or respond to a forthcoming target. The alerting network involves both cortical and subcortical areas, which share norepinephrine modulation rooted in the locus coeruleus^2,3^. The alertness state can be reached either exogenously (phasic alertness) or endogenously (tonic alertness). The former refers to a rather transient and nonspecific activation state that increases response readiness and that is triggered automatically by the presence of external warning signals (e.g., a white noise). This phasic alerting component depends on ascending thalamic projections to the right parietal lobe, which are also involved in orienting visual attention^4^. Tonic alertness develops slower and refers to the ability to maintain attention for rather long periods of time-on-task, sometimes at the scale of hours. This endogenous component is activated when the task is rather monotonous and tedious, usually because it does not require strong perceptual, cognitive, or motoric demands to be performed (examples of laboratory tasks with these characteristics are the psychomotor visual test, PVT; the continuous performance test, CPT; and the sustained attention to response task, SART). During this kind of tedious tasks, a vigilant attention network is recruited to maintain the attentional state endogenously in a top-down manner^5^. Vigilant attention involves a right lateralized cortical network including the anterior cingulate cortex (ACC) and the right dorsolateral prefrontal cortex (DLPFC), two brain areas that are also involved in executive control^6^, as well as the right inferior parietal lobe^7,8^.

Beside time-on-task, vigilant attention seems to be also affected by circadian rhythm, our endogenous biological clock that determines our physiological and behavioral processes, usually in synchrony with external time. Vigilance levels stabilize along daytime when the circadian rhythm system offsets the sleep-regulation homeostatic system, which accumulates pression to sleep as a function of time spent awake^9^. However, some people undergo shifts in circadian phase. As a consequence, individuals may differ in neurobiological functioning, such as in their peak times of day (periods of high alertness^10,11^ or vigilance^9,12^), in their sleep times, bedtime, wake up time and daytime sleepiness^13^, in their melatonin levels^14^, cortisol levels^15^, and in their body temperature rhythm^16^ and peaks^17^. All these variations are based on one’s innate circadian rhythm. In fact, people display preferences for activity at certain time of day, leading to a circadian phenotype that may be classified with the concept of chronotype. Thus, chronotype can be defined as the time of day preferred by individuals to perform daily activities or to sleep^9,18^.

Chronotype is derived from genetic factors since it depends on clock genes that interact among them determining specific circadian patterns^19^. The central coordination of circadian rhythms at an endogenous level takes place through the suprachiasmatic nucleus (SCN) of the hypothalamus, which in turn integrates external inputs such as natural and artificial light^20,21^. Despite endogenous regulation, it is important to note that humans can voluntarily modify circadian rhythms through some activities such as physical exercise, food intake or time spent resting and being awake. Such practices become inputs that modulate the circadian pattern^19^. In this sense, the efficiency of the circadian system always depends on the conjunction of physiology and behavior of the organism^20^.

These preferences for performing daily activities and resting are often determined through the use of questionnaires^22^, but also thorough some physiological indices such as body temperature or melatonin secretion, both deemed as the best markers of circadian rhythm^19^. Accordingly, three different chronotypes can be described: Morning-types (referred to as “larks”), characterized by circadian rhythmicity that occurs earlier with their optimal functioning early in the morning, Evening-types (referred to as “owls”), characterized by circadian rhythmicity that occurs later with their optimal functioning late in the evening, the two representing the two extreme typologies. The delay of Evening-types with respect to Morning-types oscillates between 2 and 4 h in the circadian rhythmicity of all variables described above^23^. Finally, a third chronotype has been referred to as the Neither-types (or intermediate), that is, those without a pronounced circadian preference^9^. Synchrony effects are frequently reported in a variety of tasks^24^, so it is possible to reach the best performance during the optimal time of day (in the morning for Morning-types, in the evening for Evening-types) compared to the non-optimal time of day (in the morning for Evening-types, in the evening for Morning-types), following the circadian typology.

As circadian variability differentially affects endogenous alertness at different times of day, it is expected that performance in tasks that require vigilant attention differs in people with extreme chronotypes depending on time of testing. Previous studies have found chronotype and time of day interactions (synchrony effect) in tasks requiring vigilant attention (e.g., the PVT)^23,25^. Besides vigilant attention tasks, synchrony effects are also expected in cognitive demanding tasks that require sustained attention to maintain an accurate level of performance, such as those involving attentional control (e.g., Stroop or flanker interference tasks), or inhibitory control (e.g., some versions of the SART^25^).

Important interactions between the two components of the alerting system have also been widely documented. For instance, top-down vigilant attention seems to compensate for the lack of sufficient arousal that monotonous and tedious tasks convey. On the other hand, the phasic component of alerting compensates for the decrement on vigilant attention as time-on-task increases. For instance, when clonidine is administered to inhibit the release of norepinephrine from the locus coeruleus, lapses of vigilant attention are observed, but they are attenuated when white noise, activating the exogenous component of the alerting network (phasic alert), is supplied while performing the task^8,26^.

The objective of the present study is threefold. As a first objective, we aimed to determine whether the high level of endogenous alert, that we assume to occur in the optimal time of day according to the individual’s chronotype, improves performance in both a rather monotonous task that is supposed to activate the vigilant attentional network (the PVT), and in a conflict demanding task that requires sustained attention throughout the task (the flanker task). Additionally, as attention is thought to be biased to novel stimulus and/or locations^27,28^, we assessed whether synchrony effects in the two aforementioned tasks can be modulated by task-related novelty, that is, first-experience-with-task occurring in the first session. It might be that first-experience-with-task increases alerting levels, compensating the low endogenous alertness when participants complete the tasks for the first time at their non-optimal time. If that were the case, we expect synchrony effects in this situation to be reduced in comparison with when the first session takes place at the optimal time of day and the second at the non-optimal time of day.

As a second objective, we aimed to determine whether the exogenous component of the alerting system modulates also people’s performance according to their chronotypes. Given the interactive relationship between the exogenous and endogenous components of alerting, we expect that warning auditory signals that are usually used to assess the effect of phasic alerting (e.g., in the interactive version of the Attention Network Test, ANT-I^29^), compensates for the low level of endogenous alertness when people perform the task at their non-optimal time of day.

Finally, as a third objective, we aimed to assess the impact of the two different components of the alerting system on executive control as a function of chronotype. Phasic alerting seems to have a negative impact in some measures of cognitive control^30–34^. For instance, conflict based on stimulus–response interference, like in the Simon task^35^ is increased when targets are preceded by an auditory signal^36^, but conflict based on stimulus-stimulus interference, like in the Stroop task is not^34^. These results suggest that alerting improves the translation of the visual code into the correspondent motor code^36^, rather than the alerting tone producing a general state of readiness to respond.

However, of greatest relevance for the present study is the negative effect of phasic alerting on flanker interference. With this task, two main accounts have been proposed. The deactivation account^29,31,37^ suggests that the alerting tone would inhibit the attentional control network involved in conflict resolution^38^, producing a subjective feeling of clearing of consciousness, and rendering the individual more prone to react to forthcoming external stimuli than to current thoughts or internal states^39,40^. Alternatively, phasic alerting might prioritize spatial processing of stimuli in the visual field, being them task-relevant or task-irrelevant, and consequently enhancing the processing of to-be-ignored distracters when they are presented separated from the target, as it happens in the flanker task^34^. Thus, the increased conflict effect with alerting warning tones would be the indirect consequence of higher accessibility of spatially presented flankers.

All these results suggest that the interaction between phasic alerting and conflict may depend on the type of interference involved. Thus, when interference is based on stimulus–response mapping, alerting may facilitate translation between visual and motor codes, whereas when interference is based on spatial processing of target and distracters, like in the present study, alerting may either inhibit the executive network or facilitate spatial processing of task-irrelevant flankers.

To elucidate which is the best explanation for the alerting and conflict network interaction is beyond the aim of the present study. However, data from synchrony effects on the conflict effect can help assess the pertinence of the two aforementioned accounts in the flanker task. According to the former explanation (clearing of consciousness), conducting the flanker task in the optimal time of day could compensate for the reduced activity of the executive control network produced by the alerting tone, as the vigilant attention network and the executive attention network share common brain areas, such as the DLPFC and the ACC^8^. Therefore, such increment in the conflict effect with the alerting tone should not be observed when participants perform the task in the optimal time of day. However, if the alerting tone increases spatial attention to distracters in the flanker task, such increment in conflict effect should be observed irrespective of the moment of day. Importantly, tonic alertness has been found to enhance spatial processing in orienting tasks^41–44^, like the phasic component does^37,45^. Therefore, according to the spatial processing account, the endogenous component of the alerting network should have similar effects on conflict effects as the alerting tone has been found to have. If that were the case, we should observe increased conflict effects when testing occurs in the optimal time of day compared with when testing occurs in the non-optimal time of day with the alerting tone absent. However, previous findings have reported no differences in flanker interference due to the endogenous component of the alerting network either when tonic alerting is promoted throughout the whole task^46^ or as a function of chronotype and time of testing^47,48^. These results suggest that the endogenous component of the alerting network has influence in spatial orientation but not in how people deal with cognitive conflict in the flanker task.

## Method

Morning-types and Evening-types participants were tested in two different sessions separated by 1 week. One session took place in the morning and the other in the evening. In each session, all participants completed two computer-based reaction-time tasks. First, the PVT, a rather monotonous task that measures vigilant attention^49^. Second, a flanker task, preceded or not by alerting tones, in which target and distracters were located in the center of the screen to avoid uncertainty about target location.

### Participants

Details of the study were announced through the distribution list existing in the Faculty of Psychology (University of Murcia) to recruit participants in exchange of course credit. Thirty-four undergraduate students (27 females; M age = 21.0 years, SD age = 2.3) were selected from a total of 132 students who agreed to participate and complete a reduced Spanish version of the Horne and Östberg’s Morningness–Eveningness Questionnaire (MEQ)^22^. On the basis of MEQ scores, we selected 16 students (14 females; M age = 21.6, SD age = 3.0) for the Morning-types group, and 18 students (13 females; M age = 20.4, SD age = 1.5) for the Evening-types group. We excluded participants with intermediate chronotype to maximize differences in vigilant attention between extreme chronotypes. We did not explore the influence of sex because only 7 participants were males. All participants reported normal or corrected-to-normal vision and no chronic medical conditions.

### Procedure

Participants first signed the written informed consent, and then completed the reduced Spanish version of the reduced Morningness–Eveningness Questionnaire (rMEQ) develop by Adan and Almirall^50^. The rMEQ consisted of five items with scores ranging from 4 (definitively Evening-types) to 25 (definitively Morning-types). Participants who scored between 17 and 25 (M = 19.4) formed the Morning-types group, and participants who scored between 4 and 11 (M = 8.6) formed the Evening-types group.

Participants were tested individually in sound-attenuated booths. All tasks were programmed in E-Prime 3 (Psychology Software Tools)^51^. Visual stimuli were presented on a 22′′ TFT monitor with a screen resolution of 1920 by 1,080 pixels. A Chronos device (Psychology Software Tools) with five buttons was used to collect responses and present auditory stimuli (via headphones).

All participants came to the laboratory twice, with an interval of seven days between the two sessions. One of the sessions was scheduled to begin at 8:00 AM (the morning session), and the other at 20:30 PM (the evening session), both lasting by 30 min of duration, approximately. This allowed us to evaluate participants in both their optimal and their non-optimal time of day according to their chronotype (when we expected their vigilant attention to be maximum and minimum, respectively). Testing was carried out during the week days, and the order of the sessions was counterbalanced across participants within each chronotype group, so that half of the participants from each group completed the first session in their optimal time and the second session in their non-optimal, with the reverse order for the other half.

Both sessions had the same structure. We began with a 5-min interview by asking participants whether they had consumed coffee during the previous two hours or other stimulants during the previous 24 h (all reported no consumption) and whether they had slept at least 5 h the night before (all reported having slept between 7 and 9 h). Then, participants performed a ten-min version of the PVT. Each PVT trial began with a random interval ranging from 2 to 10 s in which the computer screen remained black. Then, a red circle (50 pixels in diameter) appeared in the center of the screen and participants had to press, as quickly as possible, the central bottom of the response box with the index finger of their dominant hand. When the response was made, the screen went blank and a new trial began.

Immediately after the PVT, participants completed the flanker task for 15 min, approximately. The task consisted of five arrows (pointing left or right) as stimuli and alerting tones preceding half of the trials in each congruency condition. Each trial began with a fixation point (a plus sign) presented in the center of the screen for 2,500 ms. Then, a row of five arrows appeared in the center of the screen and participants indicated, as quickly and accurately as possible, whether the arrow in the middle (the target) pointed left or right by pressing the leftmost or rightmost button of the response box, respectively. The five arrows remained visible until a response was made or for 3,000 ms. In half of the trials, the four flanking arrows pointed in the opposite direction as the target (incongruent condition), whereas, in the other half, flankers and target pointed to the same direction (congruent condition). In each congruence condition, an alerting tone (a 50 ms beep of 2000 Hz) was presented prior to the target in half the trials, with a tone-target interval of 500 ms (from onset to onset). Participants completed three blocks of 72 trials with a short break between blocks. Experimental trials were preceded by 16 practice trials.

### Ethical approval

This study was approved by the Ethics Committee of the University of Murcia and was conducted conformed with the ethical standards laid down in the 1964 Declaration of Helsinki.

## Results

Data were preprocessed with R^52^, and analyzed by analysis of variance (ANOVA) with JASP 0.9.2^53^. We adopted a significance level of 0.05 for all analyses.

### Psychomotor vigilance task (PVT)

The first trial of each session was considered as practice and discarded. Besides, we considered extreme outliers and discarded reaction times (RTs) shorter than 150 ms or longer than 1,200 ms (0,11% of the data), in addition to those separated by more than six interquartile ranges from the median value of each participant in each session (an additional 0.22% of the data). We employed this lenient trimming procedure because, in order to exploit the additional information that emerges from RT distributions, and taking advantage of the relatively large number of trials per condition, we planned to perform a bin-means analysis^54,55^. Note that, with this kind of analysis, the presence of moderate outliers (as those that could result from fluctuations in alertness during a session) turns out to provide valuable information, rather than compromise data analysis and interpretation.

**Figure 1:**
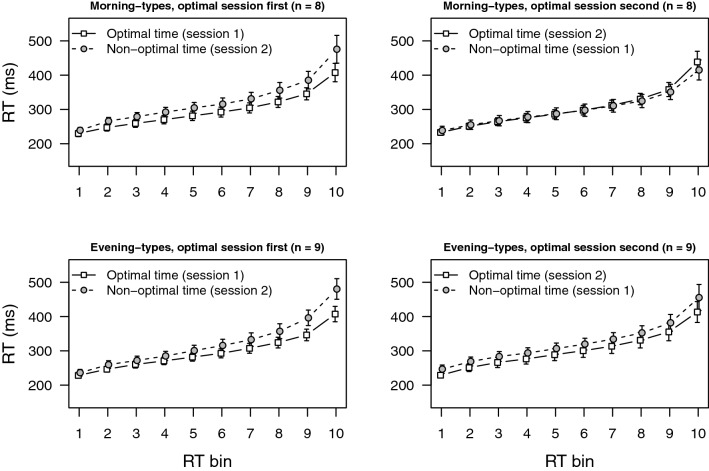
Mean reaction time (RT) in the psychomotor vigilance task, PVT (error bars represent standard error of the mean).

RTs for each participant in each session were rank ordered and divided into ten bins as equally sized as possible (the number of data per bin ranged from 8 to 11; M = 9.4). Mean RTs were calculated for each bin and submitted to a mixed analysis of variance (ANOVA) with the within-participants factors test time (optimal, non-optimal) and RT bin (1–10), and the between-participants factors chronotype (Morning-types, Evening-types) and order (optimal session first, optimal session second). Main statistical results are presented in Table 1 (rightmost column, bottom row). Neither the main effect of chronotype nor the main effect of order reached statistical significance (both *F*s < 1). However, there was a main effect of test time (the synchrony effect), revealing shorter RTs at the optimal time (M = 300 ms) than at the non-optimal time (M = 319 ms; synchrony effect = 19 ms). There was also an interaction between test time and RT bin, showing that the synchrony effect varied across bins. As illustrated in Fig. 1, the effect increased towards the slower end of the distribution (for bins 1 to 10, synchrony effect = 10, 14, 13, 14, 16, 17, 18, 21, 28, and 41 ms). Finally, there was an interaction between test time, RT bin, and chronotype; and also, between test time, RT bin, and order. To disentangle these interactions and further understand the pattern of results, we performed separate analyses for each chronotype and for each order (see Table 1). Evening-types participants showed synchrony effects that increased at the slower end of the RT distribution and that did not significantly differ as a function of session order (when the optimal session was completed first, synchrony effect for bins 1 to 10 = 8, 13, 12, 14, 19, 23, 25, 33, 50, and 73 ms; M = 27 ms; when the optimal session took place second, synchrony effect = 18, 18, 18, 17, 18, 19, 21, 22, 27, and 42 ms; M = 22 ms). This is the same pattern that was observed for Morning-types participants when the optimal session was completed first (synchrony effect = 9, 19, 20, 22, 23, 24, 26, 34, 40, 68 ms; M = 29 ms). However, the synchrony effect was completely abolished for Morning-types participants when the non-optimal session took place first (synchrony effect = 6, 4, 3, 3, 2, 0, − 1, − 6, − 7, − 24 ms; M = − 2 ms).

**Table 1:** Results of ANOVA tests on the mean reaction time (RT) for the psychomotor vigilance task (PVT).

Order	Effect	Morning-types	Evening-types	Both groups
Optimal session first	Test time	*F*(1, 7) = 7.615 *p* = 0.028, η^2^ = 0.521	*F*(1, 8) = 5.667 *p* = 0.045, η^2^ = 0.415	*F*(1, 15) = 12.843 *p* = 0.003, η^2^ = 0.461
	Test time × RT bin	*F*(9, 63) = 6.666 *p* < 0.001, η^2^ = 0.488	*F*(9,72) = 7.194 *p* < 0.001, η^2^ = 0.473	*F*(9, 135) = 13.220 *p* < 0.001, η^2^ = 0.463
	Test time × chronotype	–	–	*F* < 1
	Test Time × bin × chronotype	–	–	*F* < 1
Optimal session second	Test time	*F* < 1	*F*(1, 8) = 4.462 *p* = 0.06, η^2^ = 0.358	*F*(1, 15) = 2.119 *p* = 0.166, η^2^ = 0.105
	Test time × RT bin	*F*(9, 63) = 2.022 *p* = 0.051, η^2^ = 0.224	*F*(9,72) = 2.221 *p* = 0.03, η^2^ = 0.217	*F* < 1
	Test time × chronotype	–	–	*F*(1,15) = 3.088 *p* = 0.099, η^2^ = 0.153
	Test time × bin × chronotype	–	–	*F*(9,135) = 4.198 *p* < 0.001, η^2^ = 0.218
Both orders	Test time	*F*(1, 14) = 3.912 p = 0.068, η^2^ = 0.169	*F*(1, 16) = 10.128 *p* = 0.006, η^2^ = 0.386	*F*(1, 30) = 13.352 *p* < 0.001, η^2^ = 0.272
	Test time × RT bin	*F* < 1	*F*(9, 144) = 9.171 *p* < 0.001, η^2^ = 0.337	*F*( 9, 270) = 7.675 *p* < 0.001, η^2^ = 0.154
	Test time × Order	*F*(1, 14) = 5.218 *p* = 0.038, η^2^ = 0.226	*F* < 1	*F*(1, 30) = 3.008 *p* = 0.093, η^2^ = 0.061
	Test time × bin × order	*F*(9, 126) = 8.064 p < 0.001, η^2^ = 0.353	*F*(9, 144) = 2.041 *p* = 0.039, η^2^ = 0.075	*F*(9, 270) = 8.600 *p* < 0.001, η^2^ = 0.172
	Test time × bin × chronotype	–	–	*F*(9, 270) = 2.591 *p* = 0.007, η^2^ = 0.052

The PVT allowed us to evaluate the tonic alert that resulted from the synchrony between participants' chronotype and the time of day in which the task was performed. In addition to this form of tonic alert, the flanker task allowed us to also evaluate the phasic alert generated by the presentation of alerting tones. We also evaluated the interaction between tonic and phasic alert and the modulation of the congruency effect by these two kinds of alerting.

### Flanker task

We excluded from the RT analysis trials with incorrect responses (2.78% of the data) and trials with RTs separated by more than three interquartile ranges from the median value of each participant in each condition (1.95% of the data). Mean RTs were submitted to a mixed ANOVA with test time (optimal, non-optimal), alerting tone (tone, no tone), and congruency (congruent, incongruent) as within-participant factors; and chronotype (Morning-types, Evening-types) and order (optimal session first, optimal session second) as between-participants factors.

**Figure 2:**
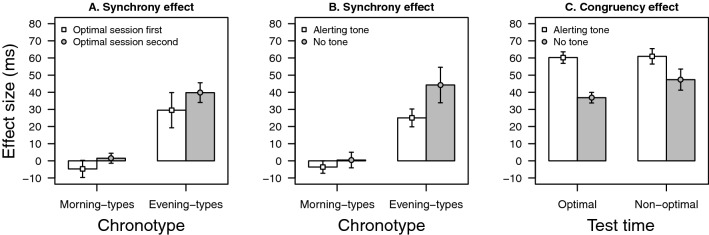
(**A**) Synchrony effect (RT at the non-optimal time minus RT at the optimal time) as a function of chronotype and order. (**B**) Synchrony effect as a function of chronotype and alerting tone. (**C**) Congruency effect (RT in the incongruent condition minus RT in the congruent condition) as a function of alerting tone and test time.

There was a main effect of test time, *F*(1, 30) = 8.212, *p* = 0.008, η^2^ = 0.169, revealing that RTs were shorter at the optimal time (M = 394 ms) than at the non-optimal time (M = 412; synchrony effect = 18 ms). However, this effect was not equivalent for Morning-types and Evening-types participants, as revealed by the interaction between test time and chronotype, *F*(1, 30) = 9.864, *p* = 0.004, η^2^ = 0.203. In fact, only the Evening-types participants showed a significant effect of test time (synchrony effect = 35 ms), whereas this effect was completely abolished in the Morning-types group (synchrony effect = − 1.6 ms). As illustrated in Fig. 2A, the synchrony effect was numerically greater when the optimal session took place second than when the optimal session was completed first, in contrast to what was observed with the PVT. However, this tendency was not statistically significant (for both the test time × order interaction and the test time × chronotype × order interaction, *F* < 1).

In addition, we found a statistically significant interaction between test time and alerting tone, *F*(1, 30) = 4.653, *p* = 0.039, η^2^ = 0.119, revealing greater synchrony effect in the no-tone condition than in the tone condition (synchrony effect = 24 and 12 ms, respectively). An inspection of Fig. 2B suggests that this interaction was mainly consequence of Evening-types participants' performance. In fact, for Morning-types participants just a main effect of alerting tone was observed, *F*(1, 14) = 31.399, *p* < 0.001, η^2^ = 0.684, that is, RTs were shorter with the tone than when the tone was not presented. However, for Evening-types participants we also observed a main effect of alerting tone, *F*(1, 16) = 23.093, *p* < 0.001, η^2^ = 0.586 but test of time and alerting tone interacted in this group of participants. Although admittedly the interaction was only marginally significant, *F*(1, 16) = 4.111, *p* = 0.060, η^2^ = 0.195 it is worth noting that the alerting tone reduced the differences between the optimal and non-optimal time of day by almost half (synchrony effect = 25 ms) in comparison to when the alerting tone was absent (synchrony effect = 44 ms).

Finally, there was statistically significant main effects of congruency, *F*(1, 30) = 233.828, *p* < 0.001, η^2^ = 0.884 and alerting tone, *F*(1, 30) = 49.415, *p* < 0.001, η^2^ = 0.614. RTs were shorter in the congruent condition (M = 377 ms) than in the incongruent condition (M = 418 ms; congruency effect = 51 ms); and RTs were also shorter in trials with alerting tone (M = 387 ms) than in trials with no tone (M = 418; alerting tone effect = 31 ms). However, the congruency × alerting tone interaction was statistically significant, *F*(1, 30) = 33.400, *p* < 0.001, η^2^ = 0.515 because the congruency effect was greater in the tone condition (congruency effect = 61 ms) than in the no tone condition (congruency effect = 42 ms). Importantly, however, the congruency × test time interaction did not reach statistical significance, *F*(1, 30) = 1.934, *p* = 0.175, η^2^ = 0.055 suggesting equivalent congruency effect at the optimal time (congruency effect = 49 ms) than at the non-optimal time (congruency effect = 54 ms; Fig. 2C).

## Discussion

Three main objectives were addressed in the present study regarding the role of chronotype in attention-related tasks. We first asked whether synchrony effects, that is, the difference in performance between the optimal and non-optimal time of day as a function of chronotype, are observed in two tasks that differently involve the endogenous component of the alerting network, the PVT and the flanker tasks. The second objective was to explore the role of phasic alerting as a function of chronotype and time of testing when participants performed the flanker task. In the final objective we assessed the role of the two components of the alerting network in executive attention-dependent conflict resolution, and whether chronotype and time of testing had any modulatory effect.

### The role of chronotype in attention tasks

The first task, the PVT, requires vigilant attention that is activated when rather monotonous tasks are performed during long periods of time. Our results showed that when participants performed the PVT at the non-optimal time of day reaction times to targets were longer than when participants performed the task at the optimal time of day^25,56^. This synchrony effect increased towards the slower end of the RT distribution, which suggests that the effect largely emerged from more frequent or extreme fluctuations of attention at the non-optimal time of day. Besides, the synchrony effect was modulated by the first-experience-with-task, but very especially for the Morning-types participants. Specifically, whereas Evening-types participants showed synchrony effects irrespective of whether the task was performed first at either the non-optimal or optimal time of day, Morning-types participants showed synchrony effects only when they performed the task first at the optimal time of day. When they performed the task first at the non-optimal time of day, the synchrony effect vanished away, being performance at their non-optimal time of day similar to that observed at their optimal time of day. This novel result suggests that novelty of the task produced an increment in the level of alerting that overcame the deleterious effect of poor vigilant attention characteristic of the non-optimal time of day, but that this novelty effect only occurred or was much more pronounced for Morning-types chronotype. Consequently, the novelty effect fully compensated the fact of being at the non-optimal time of day for Morning-types participants, but not for Evening-types participants. These results extend what is usually observed when novel objects or locations are presented along task performance^27^ to the task as a whole when it is presented for the first time under low vigilant attention conditions.

As in the PVT, Evening-types participants showed reliable synchrony effects in the flanker task irrespective of whether first-experience-with-task took place at the non-optimal or at the optimal time of day. Morning-types participants, however, did not show any synchrony effect in the two session, and first-experience-with-task did not have any modulation effect like that observed with the PVT. This unexpected pattern of results showed by Morning-types participants across tasks deserves further discussion. One possible explanation for these results is that Morning-types individuals are, in general, more sensitive than Evening-types individuals to certain factors potentially capable of increasing alerting levels at their non-optimal time of day. The observed difference between the PVT and the flanker tasks may emerge from the fact that the flanker task itself fosters sustained attention due to the high level of cognitive demands of dealing with conflicting information (the flankers), having to choose between two possible responses, and monitoring errors, whereas the PVT does not contain strong perceptual, cognitive, or motoric features that promote high level of attention. In this situation, Morning-types participants could saw their low vigilance level at the non-optimal time of day compensated by task-novelty in the PVT. In the flanker tasks, however, they reached appropriate levels of sustained attention because the high cognitive demands of the task, regardless of the session in which the task is performed.

It is not clear though, why only Morning-types participants benefited from task novelty (PVT) and cognitive demands (flanker task) to increase their alerting levels at the non-optimal time of day.

### Interaction between the two components of alerting in the flanker task

With the flanker task we also asked about the role of the exogenous component of alerting in performance as a function of chronotype and time of testing. The reduction in RTs when the alerting tone was present compared with when it was absent was observed at both the optimal and non-optimal times of day, and for both Morning- and Evening-types participants. However, in line with the previous contention, it was expected that phasic alerting compensates for the deleterious effect of reduced vigilance in the non-optimal time of day, mainly in Evening-types participants. Whereas Morning-types participants showed faster responses with warning tone present (phasic alerting effect) irrespective of time of testing, Evening-types participants showed more effect of phasic alerting when testing occurred in the non-optimal than in the optimal time of day.

These results comply with the suggestion that the phasic component of alerting, when activated, reduces the demands on the endogenous component. Let us illustrate that contention with some examples. In a fMRI study, O'Connor et al.^57^ used the SART, a task that activates the right fronto-parietal network as well as the thalamus. When participants performed the SART with auditory alerting tones being randomly presented, activation in the right DLPFC was absent. Thus, the activation of the alerting network by exogenously presented alerting tones seems to reduce the need of top-down modulation of the endogenous alerting component mediated by the right DLPFC. Also, the compensatory role of phasic alerting illustrated here, may have relevant consequences in pathology. For instance, ascending thalamic projections to the parietal lobe characteristic of phasic alerting can ameliorate parietal lobe-dependent orienting deficits shown by left-side neglect^45,46^. Lewy Bodies dementia patients showed a deficit in orienting attention, which was regulated by the presence of an alerting tone^58^. Mild cognitive impairment patients showed their conflict effect restored up to the level showed by healthy control participants when the alerting tone was present, compensating for the deficit of such patients in keeping an adequate level of tonic alertness thorough the task^59^. Finally, the fact that RTs were faster even when participants performed the task in the optimal time of day, when vigilant attention levels are high, leads us to conclude that phasic alert plays a role beyond the mere compensatory effect observed in conditions of low vigilance. Phasic alerting is thought to activate initial phases of response initiation^60^, accelerating responses sometimes at expenses of accuracy^61^.

### Effects of alerting on conflict resolution: the role of chronotype and time of testing

A final goal was to determine the role of the two components of the alerting network in executive attention-dependent conflict resolution, and whether chronotype and time of testing have any modulatory effect. As previously found, the endogenous component of alerting did not influence the congruency effect at all^46^. Tonic alertness, sustained throughout the performance of a cognitive demanding task, was sufficient to achieve an appropriate functioning of the executive attention network involved in conflict monitoring and resolution^6^, so that any further increment in vigilance, as expected in the optimal time of day, did not add any efficiency in such network functioning. Regarding the exogenous component of alerting, our results replicate those of previous studies that found an increment in the congruency effect with the alerting tone present. If phasic alerting had produced a deactivation of some components of executive attention, the increment of vigilance level usually observed in the optimal time of day, should have compensated for such reduced activity in conflict-based brain areas also involved in the vigilant attention network (e.g., ACC and right DLPFC)^8^. The fact that such deleterious effect of phasic alerting on conflict resolution was observed irrespective of participants’ chronotype and time of testing goes against a negative relationship between the alerting and the executive control networks. These results fit better with an account based on phasic alerting prioritizing spatial attention in the visual field^34^. The alerting tone might have expanded the focus of attention, enhancing processing of flankers and therefore fostering response competition with the target. As a result, the conflict effect increased. This account agrees also with the alerting tone enhancing attention orientation triggered by peripheral cues in healthy younger adults^37^.

Briefly, the exogenous and endogenous components of the alerting network interact with each other, so that phasic alerting compensates for reduced activation of the endogenous component due either to low vigilance level dependent on circadian phase, or to pathology affecting the vigilant attention network. However, executive attention-dependent conflict resolution in cognitive demanding tasks, is not affected by the endogenous component of alertness, but it is seriously compromised when the exogenous component of alerting is transiently activated, irrespective of participants’ chronotype and time of testing.

### Limitations and future directions

In line with previous related research, here we have observed that the decline in performance at sub-optimal times impacts more in Evening-types individuals than in Morning-types ones^25^, maintaining the latter more stable performance over longer periods of time at any time of day^62^. We still lack a convincing account for why Morning-types participants are more sensitive than Evening-types participants to some characteristics of the attentional tasks (novelty, cognitive demands) that compensate for their low alerting level at the non-optimal time of day. Although the number of participants in the present study is well within the range of previous related studies or even larger (see^25^) we acknowledge that insufficient power might have mainly affected our analysis of novelty effects, for which very small samples of participants in each first-experience-with-task condition were tested. Future research should address such an important issue with larger samples. Other issue is whether the pattern of results observed with extreme chronotypes generalizes to individuals of the Neither-types, which represent the 60% of the adult population^24^ or to individuals of different sex and age. For instance, during adulthood, females tend to be more morning-oriented than men^63^. Regarding age, it is well known that preadolescents and old people tend to be more morning-oriented than adolescents and young people, who are more evening-oriented^64^. Young people tend to compensate during the weekends for their sleep debt accumulated during the schooldays, producing a rather irregular sleep–wake cycle pattern, which might lead them to show misalignment between their biological and social time (social jet-lag)^65^. We suggest that the irregular circadian rhythm shown by Evening-types students attending morning classes, might affect their sleep quality, and consequently a disadvantage when testing happens in their non-optimal time of day. In contrast, Morning-types participants may show more alignment between their internal clock (circadian rhythm) and academic activities, usually starting early in the morning, rendering them more efficient in their cognitive and academic performance. Accordingly, we hypothesize that in the present study, even when Evening-types participants did not show any difference regarding sleep hours in comparison with Morning-types participants, they usually report some difficulty in waking up early to attend morning classes, which might affect sleep quality^66^, compromising academic achievement^67–69^. Future studies should be conducted to determine whether the effects of the alerting system on cognitive control as a function of chronotype generalize to individuals with either extreme or intermediate chronotypes that differ in sex and age.
